# “Pre-metastatic niches” in breast cancer: are they created by or prior to the tumour onset? “Flammer Syndrome” relevance to address the question

**DOI:** 10.1007/s13167-017-0092-8

**Published:** 2017-05-02

**Authors:** Rostyslav Bubnov, Jiri Polivka, Pavol Zubor, Katarzyna Konieczka, Olga Golubnitschaja

**Affiliations:** 1 Clinical Hospital “Pheophania”, Kyiv, Ukraine; 2 Zabolotny Institute of Microbiology and Virology, National Academy of Sciences of Ukraine, Kyiv, Ukraine; 3 Department of Histology and Embryology and Biomedical Centre, Faculty of Medicine in Pilsen, Charles University, Prague, Czech Republic; 4 Department of Neurology, Faculty Hospital Plzen, Pilsen, Czech Republic; 5 Department of Obstetrics and Gynaecology, Jessenius Faculty of Medicine, Martin University Hospital, Martin, Slovak Republic; 6 Division of Oncology, Biomedical Centre Martin, Jessenius Faculty of Medicine, Comenius University in Bratislava, Martin, Slovak Republic; 7 Department of Ophthalmology, University of Basel, Basel, Switzerland; 8 Radiological Clinic, Rheinische Friedrich-Wilhelms-Universität Bonn, Sigmund-Freud-Str 25, 53105 Bonn, Germany; 9 Breast Cancer Research Centre, Rheinische Friedrich-Wilhelms-Universität Bonn, Bonn, Germany; 10 Centre for Integrated Oncology, Cologne-Bonn, Rheinische Friedrich-Wilhelms-Universität Bonn, Bonn, Germany

**Keywords:** Predictive preventive personalised medicine, Breast cancer, Metastatic disease, “Seed and Soil” theory, Liver, Flammer syndrome, Systemic hypoxia, Patient stratification

## Abstract

Breast cancer (BC) epidemic in the twenty-first century is characterised by around half a million deaths and 1.7 million new cases registered annually worldwide. Metastatic disease is the major cause of death in BC patient cohorts. Current statistics are much alarming from the viewpoint of the early mortality amongst BC patients with de novo metastatic disease. A new paradigm of so-called “pre-metastatic niches” may sufficiently promote our knowledge regarding potential pathomechanisms, individual predisposition and prognosis in development and progression of the metastatic disease. However, the crucial question remains unaddressed, whether hypoxic pre-metastatic niches in BC are created by or prior to the tumour onset. So far, the current interpretation of the “Seed and Soil” theory of metastasis proposing that the pre-metastatic niches are formed by primary tumours which “induce and guide” the process is incomplete, since it does not provide satisfactory explanations towards several facts overviewed in the article. The overall results of this study clearly support the working hypothesis presented by the authors proposing that the epi/genetic predisposition of individuals at risk to form the systemic hypoxic pre-metastatic niches can be established a long time before breast malignancy is clinically manifested. “Flammer Syndrome” (FS) phenotype may strongly contribute to particularly poor outcomes of metastatic breast cancer. Significance and relevance of individual FS symptoms for breast cancer metastatic disease are discussed in extenso.

## Introduction

Breast cancer (BC) epidemic in the twenty-first century [1] is characterised by around half a million deaths and 1.7 million new cases registered annually worldwide [2]. Metastatic disease is the major cause of death in BC patient cohorts. The lymph nodes, bones, lung, liver and brain are the most frequently reported sites of metastatic disease in BC. For example, over 20% of the entire patient cohort suffering from aggressive metastases in the liver are individuals with primary tumours diagnosed in the breast [3]. Further, the brain is one of the predominant sites of metastatic disease recorded for more than 20% of some specific BC subgroups [4, 5], although in BC-free populations primary brain tumour is a rare disorder. This phenomenon has not been yet adequately explained, but potential solutions have been well addressed in the recently published article “Mystery of the Brain Metastatic Disease in Breast Cancer Patients: Improved Patient Stratification, Disease Prediction and Targeted Prevention on the Horizon?” [6]. Current statistics are much alarming from the viewpoint of the early mortality registered amongst BC patients with de novo metastatic disease. Hence, the issue-dedicated study recently performed in the USA has demonstrated that 15.9 and 33.2% of patients died within the first 4 weeks and 6 months of the diagnosis, respectively, in 2000; 13.4 versus 26.3% of patients died within the same time frames in 2011 [7]. Moreover, the overall situation is even more dramatic in some specific subgroups such as the triple-negative BC with more than 50% of patients who died within the first 6 months of the metastatic BC diagnosis [7]. In contrast to the lowest prevalence in Luminal A (2%), the highest prevalence (12%) of the local recurrence and the highest rates of distant metastases (27.4%) have been reported for the triple-negative BC followed by HER-2-positive (19.2%), Luminal B (12.1%) and Luminal A (6.4%) subtypes [8]. Contextually, triple-negative BC patients being slim (BMI <18.5) and lymph node-positive demonstrate particularly poor overall survival rates [9]. Further, specifically young (≤35-year-old) BC patients with metastatic disease demonstrate significantly lower 5-year disease-free survival and significantly higher prevalence of distant metastasis against elderly patients (≥65 years old) [10].

A new paradigm of the so-called “pre-metastatic niches” may sufficiently promote our knowledge regarding potential pathomechanisms, individual predisposition and prognosis in development and progression of the metastatic disease. The main concept is that, in order to get effectively “domesticated” and finally colonise within distant organs, circulating tumour cells (CTCs) spread by the initial cancer need a “fertile” microenvironment which means the “pre-metastatic niches”. Many papers are dedicated to the cellular and molecular biological characteristics of the local microenvironment demonstrating a specific cellular make-up within the pre/metastatic sites of the hosting organ [11], essential involvement of exosomes [12] and the extra-cellular matrix (ECM) components [13]. Specifically, the hypoxic tumour microenvironment is considered the driving force for breast cancer progression [14, 15]; consequently, the drugs inhibiting hypoxia-inducible factor have been proposed to treat triple-negative BC patients who demonstrate particularly poor outcomes as described above.

However, the crucial question remains unaddressed in the current scientific literature, whether hypoxic pre-metastatic niches in breast cancer are created by or prior to the tumour onset. So far, the interpretation of the “Seed and Soil” theory of metastasis [16, 17] proposing that the pre-metastatic niches (soil) may be formed by primary tumours (seeds), which “induce and guide” the process [18, 19], is incomplete, since it does not provide satisfactory explanations towards the following facts. Firstly, there is quite a number of clinical cases with distant metastases diagnosed prior to a detection of primary breast tumours; the latter remain undiagnosed by standard screening programmes as recently discussed in the article “Feeling Cold and Other Underestimated Symptoms in Breast Cancer: Anecdotes or Individual Profiles for Advanced Patient Stratification?” [20]. As described above, specifically triple-negative BC is characterised by particularly aggressive metastatic disease frequently developed in parallel to the primary tumour appearance. Secondly, considering breast malignancy as a kind of “artificial” organ which, according to the current paradigm, “manipulates” gene expression patterns systemically, the tumour size should be essentially taken into consideration. It seems to be quite unrealistic, from the viewpoint of the physical and chemical capacity of the microscopically small tumours to be able to saturate the entire blood stream (5–7 l) with a so large quantum of gene products which are capable of changing the genetic programme of incomparably larger and distant organs so that they start to create pre- and metastatic niches.

## Working hypothesis

Here, we hypothesise a strong epi/genetic predisposition of individuals at risk to form the “fertile” hypoxic environment and systemic pre-metastatic niches a long time before breast cancer is clinically manifested. According to our hypothesis, the specific phenotype of the “Flammer Syndrome” (FS) might be particularly stimulating for the metastatic disease by forming the systemic hypoxic environment as described earlier [20]. To this end, the specific symptoms of the FS have been described for several populations [21], pathologies [22–26] and healthy individuals [27].

The relevance of the specific FS symptoms for BC pathology has been recently demonstrated [28]. BC-relevant symptoms of the FS, such as deficient thermoregulation, feeling inadequately cold, altered sensitivity to different stimuli, altered sleep patterns, tendency towards headache, migraine attacks and dizziness, amongst others, are assumed to be highly relevant for the metastatic disease as well. In order to verify our hypothesis, the reported here multi-centred study investigates the prevalence of FS symptoms in patients with metastatic BC versus BC-free individuals.

## Materials and methods

### Flammer Syndrome diagnostic approach

The Flammer Syndrome (FS) phenotype has been characterised earlier [29]. The FS questionnaire applied to the actual study has been developed at the University Hospital Basel, Switzerland. The actual version of the FS questionnaire has been successfully applied to study different populations [21] and FS symptoms in retinitis pigmentosa [24], as well as in multiple sclerosis [23] and other clinically relevant patient cohorts [25, 26]. Consequently, the actual version of the FS questionnaire has been used by several oncological centres involved in our current breast cancer dedicated pilot project [28].

### Experimental design of the multi-centred study

**Fig. 1:**
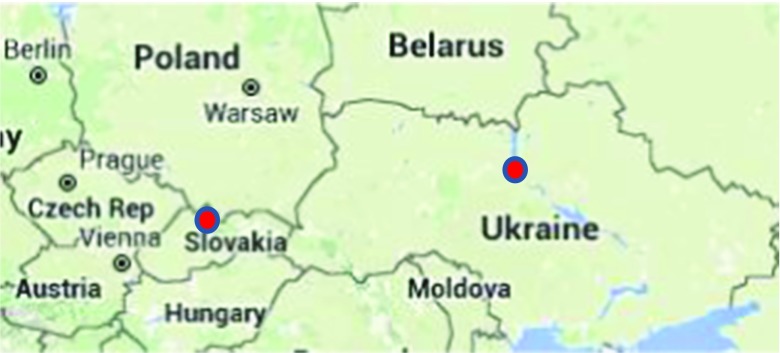
The specialised medical centres in Slovakia and Ukraine (marked with *red points*) networked by this multi-centred project are demonstrated on the map within the European context. Slovakia and Ukraine are situated in the direct neighbourhood. Both countries border to Poland and Hungary. Populations of both countries demonstrate cultural similarities to each other

Patient cohorts recruited at the specialised medical centres—Clinical Hospital “Pheophania”, Kyiv, Ukraine, and Department of Obstetrics and Gynaecology, Jessenius Faculty of Medicine, Martin University Hospital, Martin, Slovak Republic—were involved in this multi-centred study. Thereby, the patient collective comprising breast cancer-free individuals (“BC-free reference” patient cohort) has been created in Slovakia, and the cohort of patients suffering from the metastatic breast cancer has been selected and investigated in Ukraine. In Fig. 1, both centres are marked on the map within the European context. Both centres, in a tight collaboration with other partners of the international pilot project, have elaborated including and excluding criteria for the current study and followed the same norms of ethics in accordance with the ethical standards of the institutional and national research committees and with the international principles of the 1964 Helsinki Declaration and its later amendments.

One part of the patients have been selected in the databases of the centres involved and then contacted telephonically by authorised persons who have explained the meaning/rules of the study and interviewed the responders through the entire questionnaire. The other part of the patients have been personally contacted and interviewed during their stay at the centres involved.

### Breast cancer-free reference cohort

The patient database available at the Department of Obstetrics and Gynaecology, Jessenius Faculty of Medicine, Martin University Hospital, Martin, Slovak Republic, has been utilised for selecting the breast cancer-free individual patients as potential responders for the above noted FS questionnaire. The entire methodology has been described in the original article [26]. The following diagnoses/patients have been chosen as the including criterion:

- Healthy individuals free of breast cancer and any other malignancy;
- Benign breast fibroadenoma (FIA) patients, free of breast cancer and any other malignancy.

Altogether, 73 breast cancer-free (21 FIA and 52 healthy) individuals have been recruited for this study.

#### Healthy individuals

All these individuals have been clinically examined attesting an absence of gynaecological problems and interviewed personally for the study during their hospital outpatient visit performed in the framework of the national screening programme. BC-free condition has been confirmed either by breast sonography or mammography or both imaging approaches. The examination reports used were not older than 6 months. Healthy individuals demonstrated no history of any previously diagnosed breast pathology, no surgery performed due to breast lesions and no history of any severe gynaecologic disease including cancer other than breast malignancies or any systemic diseases such as diabetes mellitus, rheumatic diseases and neurological disorders.

#### Fibroadenoma patients

Imaging technologies have been applied for the 1st choice of the entire diagnostic procedure: digital mammography Hologic system, 2D + 3D sonography Voluson E8 USG system and E10 machine, BI-RADS 0–6 classification scoring system, with double reading of the radiologic approach. In case of reasonable suspicion, the affected patients have undergone biopsy analysis (either core needle or Mammotome’s vacuum-assisted). Histopathological analysis described for this multi-centred study earlier [28] has, further, allowed for distinguishing between BC malignancy and FIA benignancy. FIA individuals have been included into the BC-free patient pool.

### Metastatic breast cancer patient cohort

The patient database available at the clinical hospital “Pheophania”, Kyiv, Ukraine, has been utilised for selecting the metastatic breast cancer patients who underwent outpatient consulting diagnosis and treatment in the time frame from November 2015 till January 2017. Inclusion criteria are as follows: metastatic, locally advanced, inflammatory-edematous and recurrent breast cancer. Exclusion criteria are as follows: chronic infectious disease (HIV, hepatitis, etc.), rheumatic and neurologic diseases and thyroiditis. All the selected metastatic BC patients underwent general clinical and biochemical examinations according to the standard protocols approved by the Ministry of Health in Ukraine (Governmental Directive Number 396 titled “On the Approval and Implementation of Medical and Technological Documents for Standardisation of Medical Care of Breast Cancer” issued on 30 June 2015) [30] conform with the European and international unified protocols for primary, secondary (specialised) and tertiary (highly specialised) medical care of breast cancer patients. Extensive investigations of metastatic lesions have been performed utilising the medical imaging by sonography including also thyroid, abdominal, pelvic and musculoskeletal examinations for diagnostic purposes such as to clarify potential vascular dysregulation, hormonal and oncology-related pathologies, amongst others. For diagnostic clarifications, additional examinations have been performed by magnetic resonance imaging, computer tomography and skeletal radioscintigraphy upon individually recognised necessity.

In order to avoid any misconduct and misinterpretations, a highly individualised approach was taken for the doctor-patient communication explaining the purposes of the study prior to interviewing the patients towards the FS questionnaire. The questionnaire items have been carefully discussed during each individual interview, to avoid potential bias, and if suspicious regarding the clarity, the corresponding question was asked again for the final clarification of the correct answer, which the patient was best satisfied with, carefully choosing between “no”, “yes” (“frequently” versus “sometimes”) and “I do not know”. It should be essentially noted that the responses given towards individual questions refer to the health condition and behaviour of the patients considered in a long-term manner but not restricted to the period of time dedicated to the treatment of breast cancer and metastatic disease.

### Statistical analysis

For analytical and statistical evaluations, the collected data have been transferred to Microsoft Excel. SPSS Statistics v20.0.0 software (IBM, Armonk, New York, USA) has been applied. The prevalence of individual symptoms in groups of comparison has been evaluated and expressed in percentages. Pearson’s chi-square test of associations has been applied. *p* values below 0.05 have been considered as statistically significant.

## Results

### Statistics for the age and menopausal status in groups of comparison

Table 1 presents statistics provided for the group of breast cancer patients (27 of total) versus the group comprising BC-free individuals (73 of total) as well as for breast cancer subgroups subdivided according to their menopausal status. Premenopausal patients created the youngest group, the mean age (49 years) of which is, therefore, similar to that of the BC-free reference group (50 years). A substantially older group was created by postmenopausal BC patients. However, the difference between the age mean values has been found statistically non-significant.

**Table 1:** Age and menopausal status statistics for the groups of comparison: breast cancer patients (27 of total) and breast cancer-free individuals (73 of total)

BC menopausal status/number of patients	Premenopausal BC	Postmenopausal BC	BC total	BC-free Ref/number of patients
	13	14	27	73
Patients’ age: mean value (min–max), in years	49 (37–56)	61.38 (52–71)	56 (37–71)	50.19 (19–89)

Breast cancer patients have been subdivided into two subgroups, namely premenopausal (13 patients) and postmenopausal (14 patients). Age mean difference is statistically non-significant

### General parameters of the metastatic BC patient group investigated

Description of the metastatic breast cancer in the patient cohort investigated by the current study is summarised in Table 2. Noteworthy, all the premenopausal BC patients demonstrated liver metastasis; the same is true for the postmenopausal BC patients with one exception. The noticeable premenopausal patient marked in yellow is discussed as the “Case 1” within the “Results”, section dedicated to the “Metastatic BC—selected cases”.

**Table 2:**
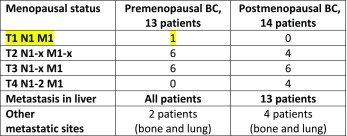
Description of the metastatic breast cancer in the patient cohort investigated by the current study

Noteworthy, all the premenopausal BC patients demonstrated liver metastasis; the same is true for the postmenopausal BC patients with one exception. The noticeable premenopausal patient marked in yellow is discussed in more detail in the main text of the “Results” section

### FS prevalence evaluated by individual symptoms

**Fig. 2:**
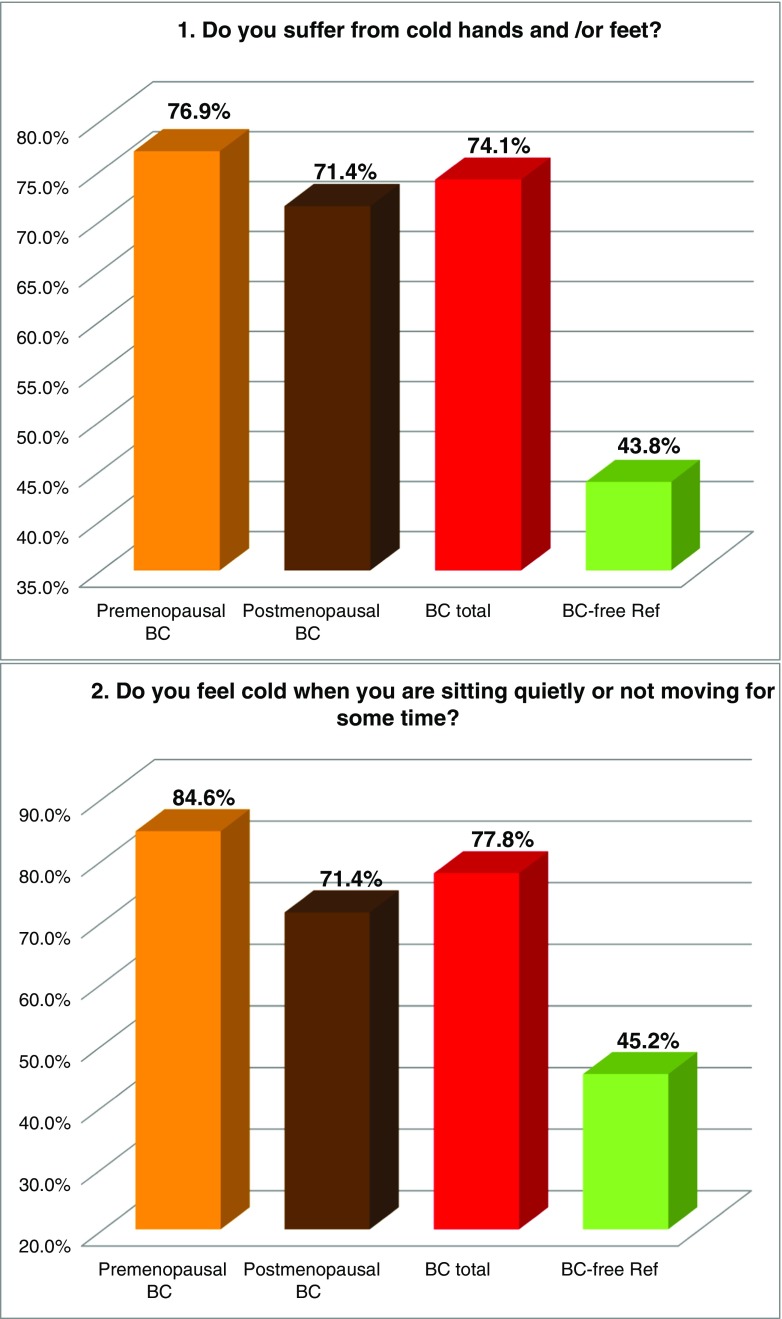

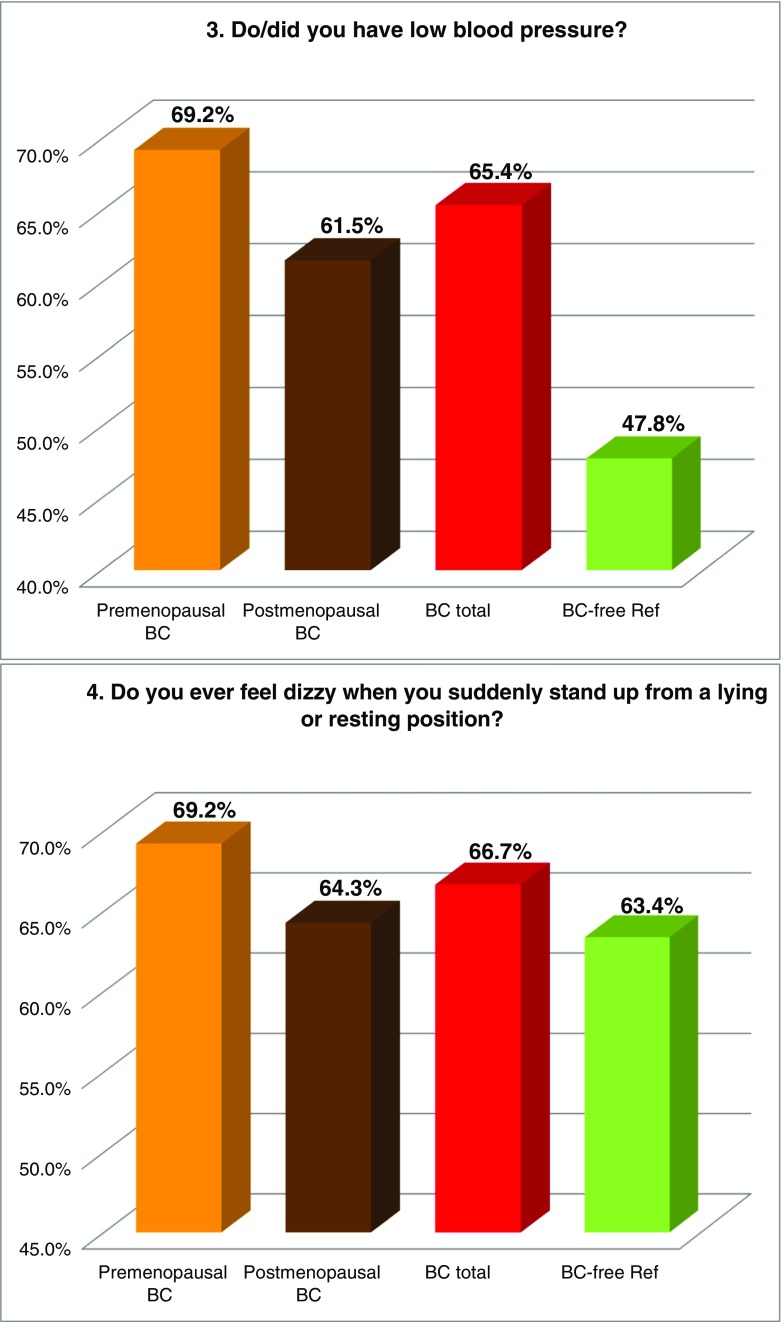

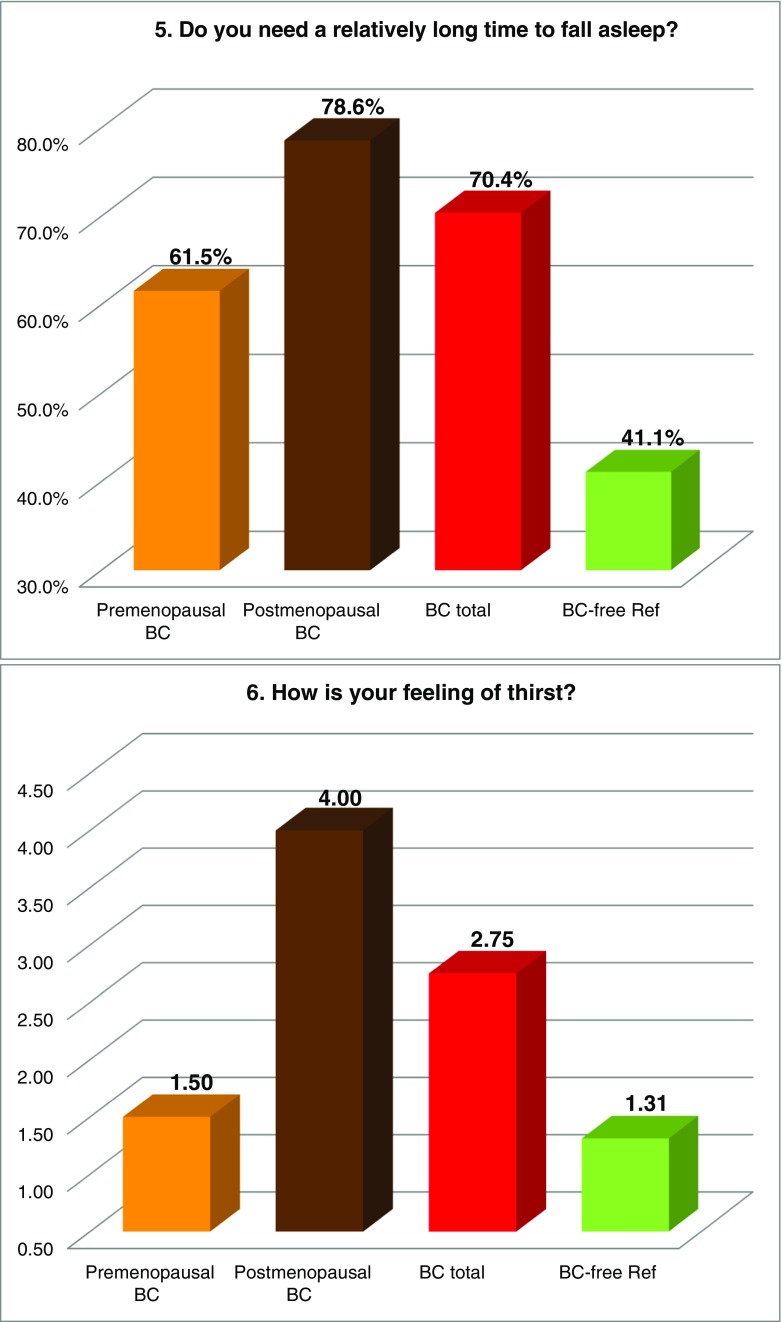

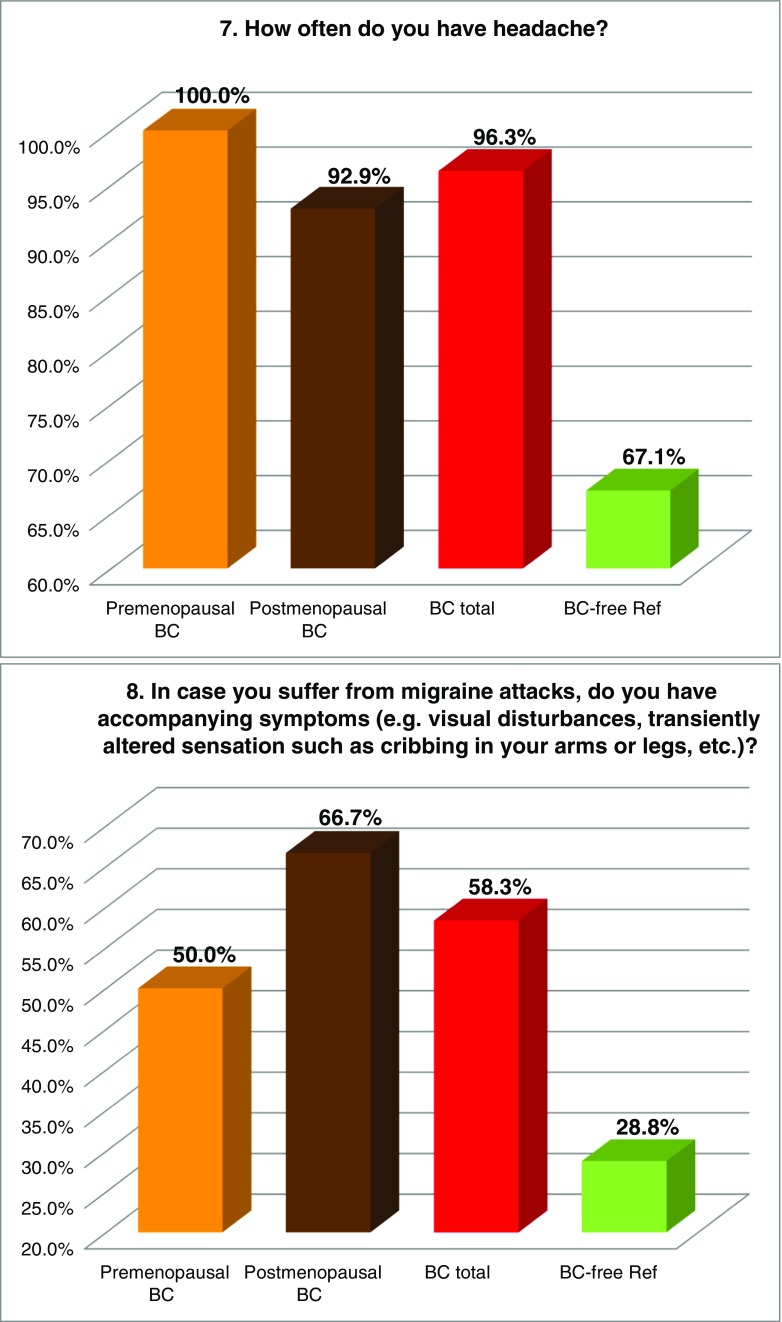

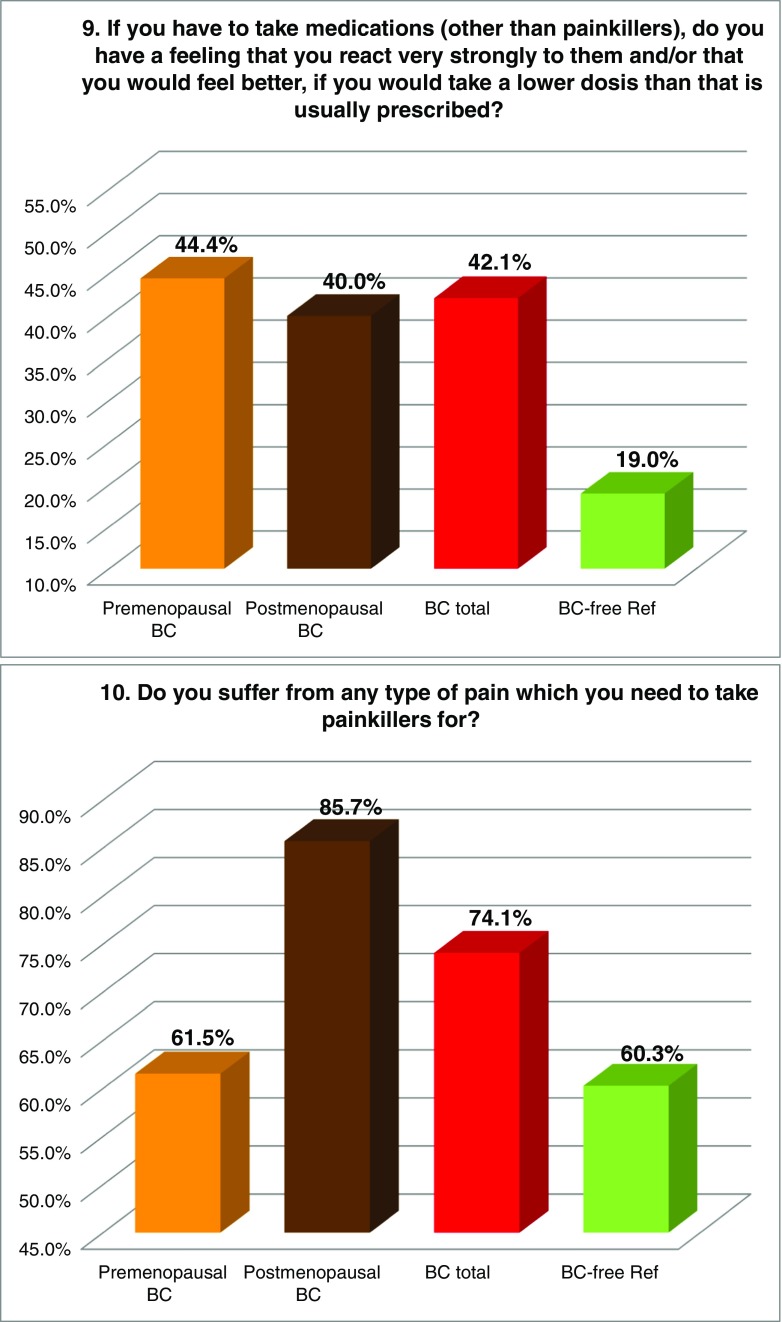

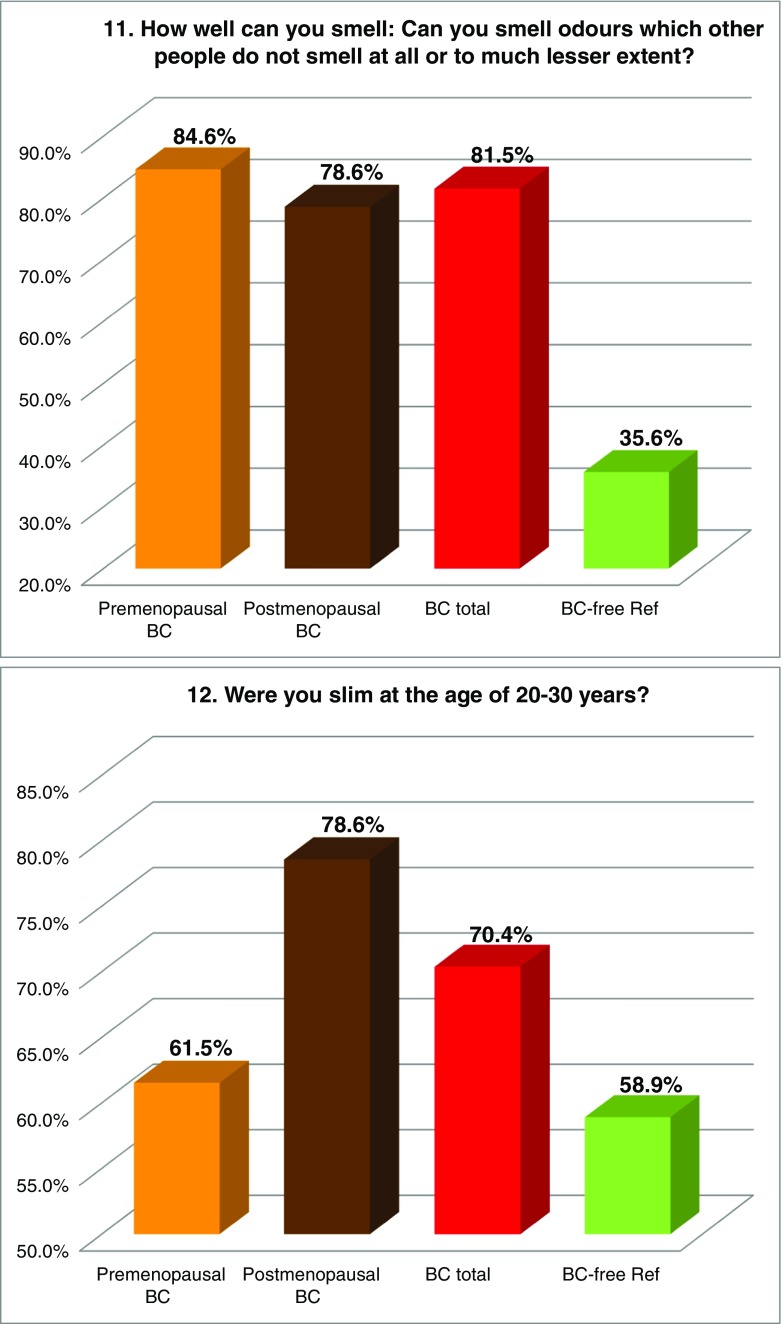

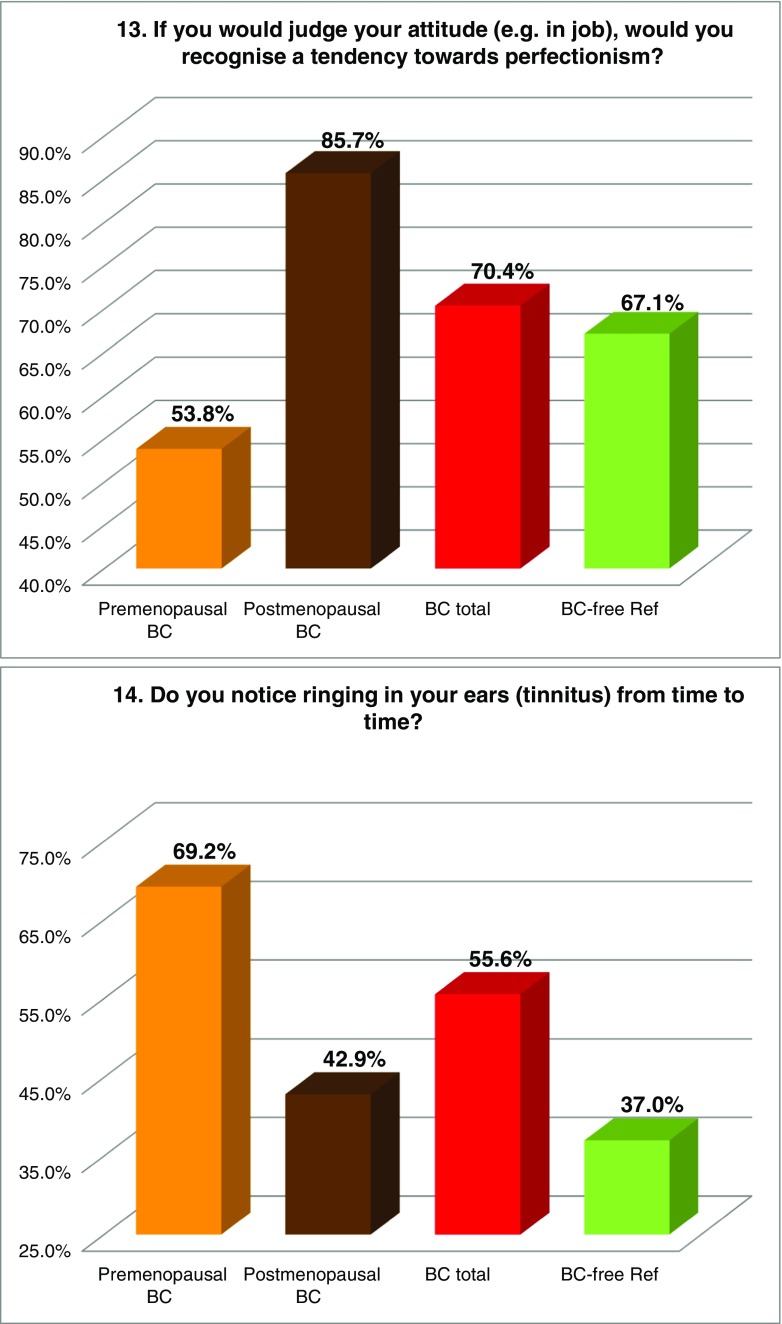

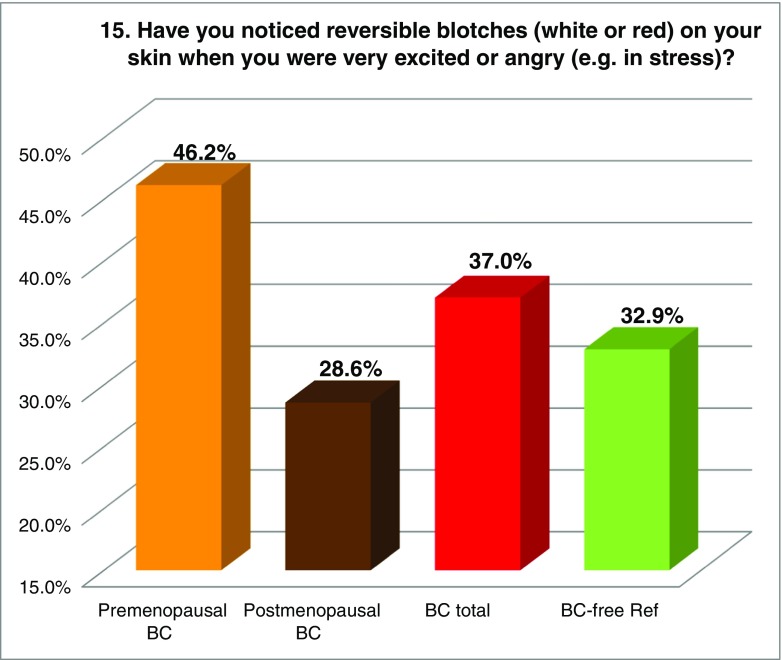
Evaluation of the prevalence of individual symptoms (1–15) of the “Flammer Syndrome” phenotype in two groups of comparison: “Breast cancer diseased” (BC total) versus “Breast cancer-free” reference (BC-free Ref) groups. Therein, the entire breast cancer patient pool (“BC total”) has been additionally analysed in subgroups stratified according to the menopausal status of the patients. For more details regarding the patient’s recruitment and stratification, see “Materials and methods” section. The prevalence in each individual group is presented by percentage of individuals who have responded to the corresponding question with “frequently” and “sometimes” pooled together. Responders answering with “I do not know” have been excluded from the overall numbers/calculations. Question-specific notes: question 6—the ratio between “I do not feel thirsty and drink little” and “I feel much thirsty and drink a lot” has been calculated and expressed as X times; question 12—answers “very slim” and “slim” are pooled together and presented in percentage

Figure 2 summarises the prevalence of individual “Flammer Syndrome” symptoms (1–15) in two main groups of comparison—“BC total” patients versus the reference group of “BC-free” individuals as well as in individual subgroups of BC patients subdivided according to their menopausal status. Higher prevalence in “BC total” (marked in red) has been demonstrated for all 15 symptoms investigated in this study. Statistical significance has been recorded for symptoms 1 (cold extremities), 2 (feeling cold), 5 (prolonged sleep onset), 7 (headache), 8 (accompanying symptoms), 9 (drug sensitivity) and 11 (smell perception); corresponding *p* values are provided in Table 3. Although being statistically non-significant (*p* = 0.103), a substantially greater prevalence has been demonstrated for symptom 6: “no feeling of thirst and drinking too little” the BC patients, in general, have demonstrated two times more frequently compared to the disease-free reference group; for the postmenopausal BC, this difference was even more pronounced. Also, the appearance of tinnitus (symptom 14), although being statistically non-significant (*p* = 0.095), was evidently more frequent in BC, particularly in the premenopausal subgroup demonstrating about two times higher prevalence compared to the disease-free reference group. Symptom 3 (low blood pressure) was more specific for the premenopausal BC demonstrating 22% higher prevalence against the disease-free reference group. In contrast, symptom 12 (low body weight in early adulthood) was more specific for postmenopausal BC. A slightly higher prevalence was demonstrated for symptom 4 (dizziness) in BC. Strong plurality has been demonstrated amongst the BC subgroups for the following three symptoms: 10 (pain, more specific for the postmenopausal BC), 13 (perfectionism, more specific for the postmenopausal BC) and 15 (skin blotches in stress, more specific for the premenopausal BC) as summarised in Table 3.

**Table 3:**
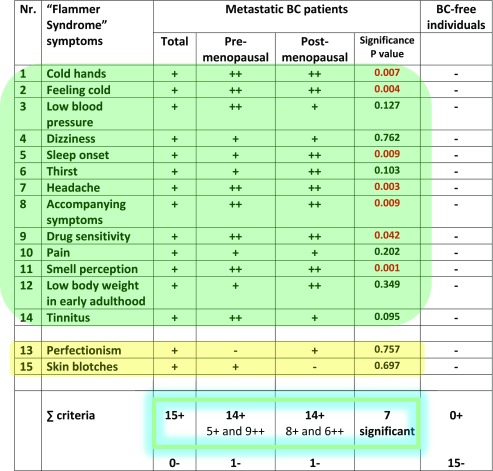
Symptoms of the “Flammer Syndrome” recorded in “Metastatic breast cancer patients” versus “Breast cancer-free individuals” groups of comparison

The table summarises the results demonstrated in Fig. 2; the following system is employed: “+” means higher prevalence of the corresponding symptom (above the lowest average of the groups of comparison); “−“ means lower prevalence of the corresponding symptom (lowest average and below it); “++“ means values sufficiently over the highest average. All 15 symptoms demonstrate increased prevalence in BC total versus BC-free. The level of significance is noted: *p* values below 0.05 are considered statistically significant and marked in red colour (symptoms 1, 2, 5, 7, 8, 9, 11). Thirteen symptoms united within the green-marked cluster demonstrate the prevalence ultimately increased in BC total as well as BC subgroups. Although the prevalence of symptoms 13 and 15 (yellow-marked cluster) is slightly increased in “BC total” compared to “BC-free,” it varies in BC subgroups demonstrating a particularly strong plurality amongst the patients with the metastatic BC investigated in the current study

### Metastatic BC—selected cases

#### Case 1

**Fig. 3:**
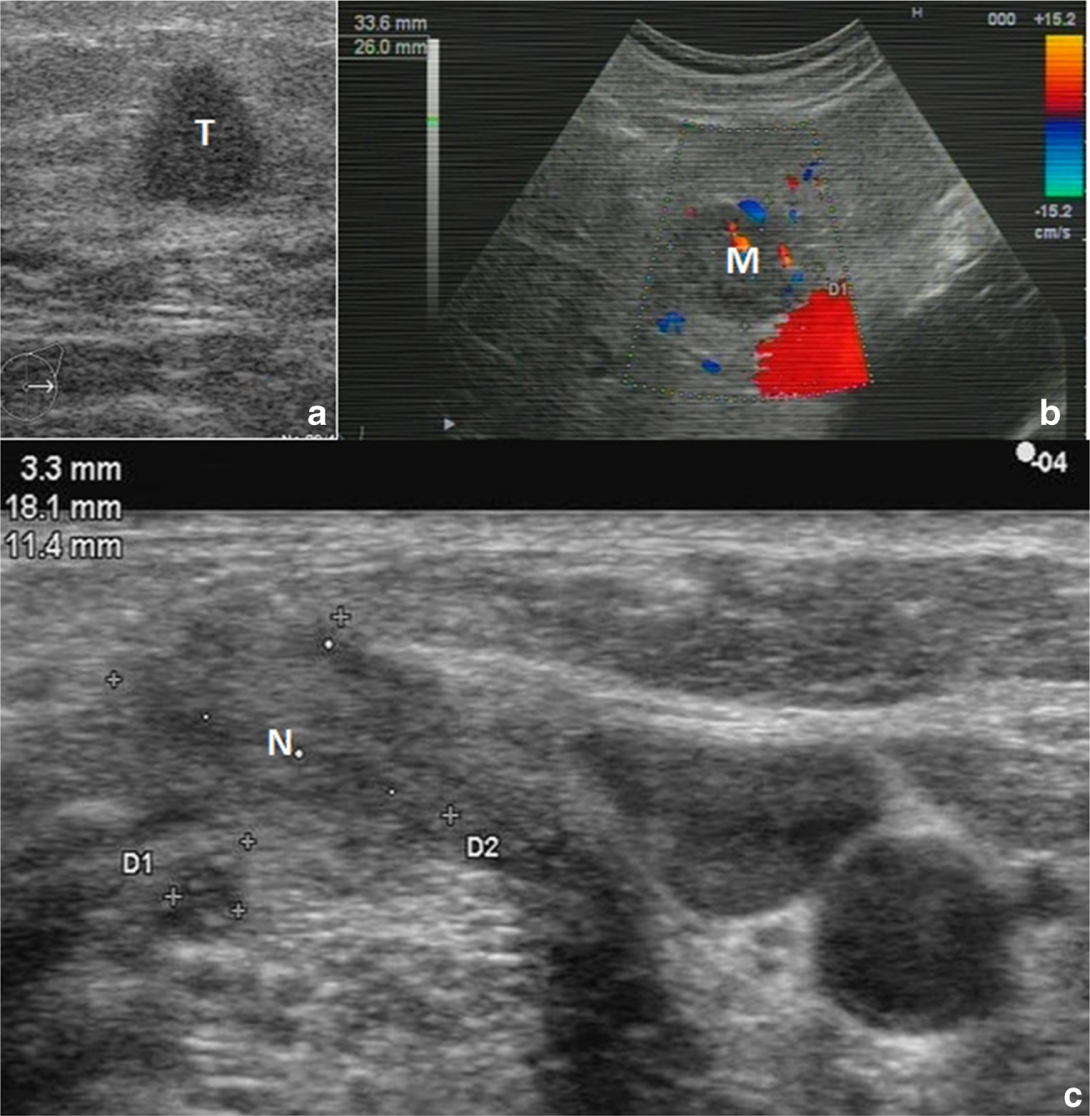
Medical imaging by ultrasound illustrating the “Case 1” (within the “Metastatic BC—selected cases” section) of the patient diagnosed with the metastatic breast cancer T1N1M1; for the exact description of this case and its relevance for the “Flammer Syndrome”, please see “Results” section. **a** Small initial tumour in the breast (T). **b** A metastatic lesion in the liver (M). **c** A metastasis in the axillary lymph node (N)

This patient is marked in yellow colour within Table 2. Corresponding medical imaging by ultrasound examination is demonstrated in Fig. 3. A female breast cancer patient, premenopausal, aged 48 years, T1N1M1, with a small tumour (below 1 cm of size) initially detected in the breast followed by multiple-site secondary metastases detected in the bone and liver, has been interviewed for FS symptoms. The interview resulted in 13 positive responses from the maximum of 15 (see “Materials and methods” section). A negative response was given regarding symptom 5 (answered as “rather normal sleep onset”); further, the patient replied as “I do not know” regarding symptom 9 (drug sensitivity). Particularly noticeable responses have been given towards the following symptoms:

- Symptom 2—feeling cold frequently
- Symptom 8—strong migraine attacks and frequently observed accompanying symptoms such as an impaired vision, deafness appeared in the extremities, etc.
- Symptom 11—strongly pronounced smell perception
- Symptom 12—slim body shape in early adulthood
- Symptom 13—strongly pronounced tendency towards perfectionism
- Symptom 15—evident skin blotches in stress situations

#### Case 2

A female patient, postmenopausal, aged 60 years, BMI = 17.9, was diagnosed with left breast cancer in 2014, Т4N1М1, and in 2016 with aggressive metastatic disease in liver, lung and bone, despite the chemotherapeutic treatment. The patient has been interviewed for FS symptoms. The interview resulted in 9 positive responses from the maximum of 15 (see “Materials and methods” section). The negative responses were given regarding symptoms 3 (low blood pressure), 9 (drug sensitivity), 14 (tinnitus) and 15 (skin blotches). The body shape in early adulthood corresponded rather to the average (symptom 12). No answer was provided for 8 (accompanying symptoms). Particularly noticeable responses have been given towards the following symptoms:

- Symptom 1—frequently cold extremities
- Symptom 2—frequently feeling cold
- Symptom 5—evidently prolonged sleep onset
- Symptom 6—“do not feel thirsty and drink too little”
- Symptom 7—frequent headache
- Symptom 13—strongly pronounced tendency towards perfectionism

#### Case 3

A female patient, postmenopausal, aged 62 years, was diagnosed with Paget’s disease of the left breast (Т1N0М0) in 2014, and in 2016 with aggressive metastatic disease in the liver and bone, despite chemotherapeutic treatments. Collateral diseases are angina pectoris, myocardial infarction and heart failure. The patient has been interviewed for FS symptoms which resulted in 12 positive responses from the maximum of 15 (see “Materials and methods” section). Negative responses were given regarding symptoms 9 (drug sensitivity) and 14 (tinnitus). No answer was provided for 8 (accompanying symptoms). Particularly noticeable responses have been given towards the following symptoms:

- Symptom 1—frequently cold extremities
- Symptom 2—sometimes feeling cold
- Symptom 4—frequent dizziness
- Symptom 5—evidently prolonged sleep onset
- Symptom 6—“do not feel thirsty and drink too little”
- Symptom 7—frequent headache
- Symptom 10—frequent pain
- Symptom 11—strongly pronounced smell perception
- Symptom 12—slim body shape in early adulthood
- Symptom 13—strongly pronounced tendency towards perfectionism
- Symptom 15—evident skin blotches in stress situations

#### Case 4

**Fig. 4:**
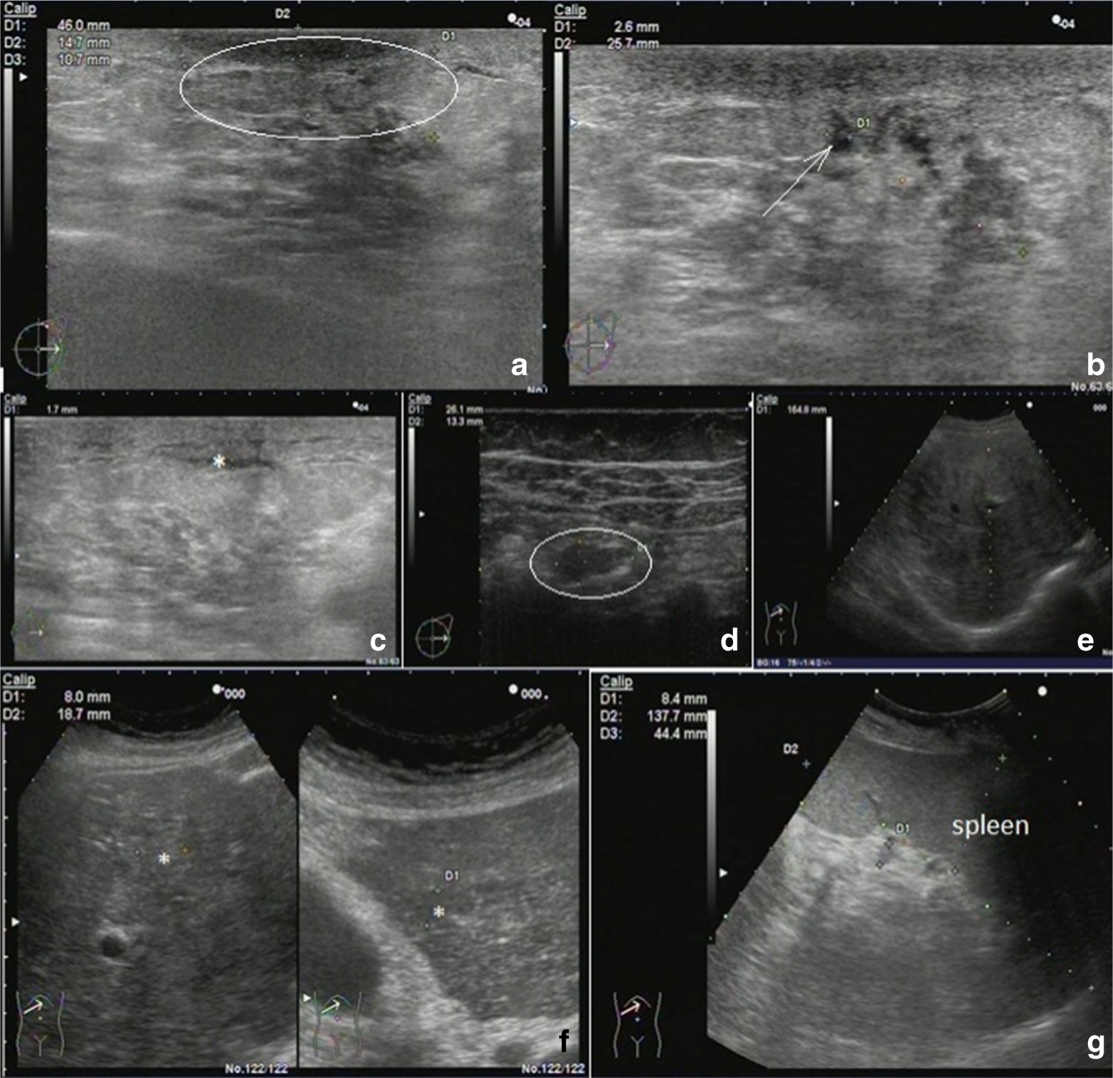
Medical imaging by ultrasound illustrating the “Case 4” (within the “Metastatic BC—selected cases” section) of the patient diagnosed with metastatic breast cancer T3N1M1; for the exact description of this case and its relevance for the “Flammer Syndrome”, please see “Results” section. **a** Left breast subareolar lesion (*ellipse*). **b** Ductal ectasia (*arrow*). **c** Signs of lymphostasis (*asterisk*); **d** Axillar lymphatic node (*ellipse*). **e**, **f** Diffuse multiple metastatic liver lesions (*asterisk*), intra-hepatic cholestasis. **g** Splenomegaly which is a usual sign of portal hypertension; notable: the liver parenchyma is diffusely (subtotally, in all segments) mottled by the merging multiple iso-, hypo-, and hyper-echoic lesions (*arrows*). Intra-hepatic bile ducts are dilated up to 2–3 mm. The liver impairment and cholestasis known by their synergistic effects belong to the collateral pathologies specifically leading to poor prognosis in metastatic breast cancer outcomes

A female patient, postmenopausal, aged 58 years, BMI = 20.8, was diagnosed with metastatic breast cancer (Т3N1М1, liver metastasis), followed by aggressive metastatic disease in bone and lung, despite the chemotherapeutic treatments. Corresponding medical imaging by a complex ultrasound examination is demonstrated in Fig. 4. The patient has been interviewed towards FS symptoms that resulted in 14 positive responses from the maximum of 15 (see “Materials and methods” section). The negative response was given against symptom 6, answered as “I feel thirsty and drink a lot”. Particularly noticeable responses have been given towards the following symptoms:

- Symptom 1—frequently cold extremities
- Symptom 2—frequently feeling cold
- Symptom 11—strongly pronounced smell perception
- Symptom 12—slim body shape in early adulthood
- Symptom 13—strongly pronounced tendency towards perfectionism

## Discussion

The particular advantage of this study is the highly personalised management of the “doctor-patient” communication for interviewing the patients involved. As described in the “Materials and methods” section, all the questionnaire items have been carefully discussed during each individual interview, to avoid any potential misinterpretation and to fix the answer, which the patient was best satisfied with. Consequently, the results reported are well reliable and provide a robust scientific platform for the data analysis, presented concepts and follow-up projects expanding for higher numbers of the patients to be involved.

Further, it was our ultimate intension, along with the general statistics collected for the entire groups of comparison, to provide a spectrum of case reports. This spectrum demonstrates on one side highly individual parameters of every patient and on the other side a great consensus for all of them regarding both the disease severity and corresponding patterns of the FS symptoms.

The overall results of this study clearly support the working hypothesis presented by the authors proposing that a strong epi/genetic predisposition of individuals at risk to form the systemic hypoxic pre-metastatic niches can be established a long time before breast malignancy is clinically manifested. The FS phenotype may act over a couple of years or even several life-decades as a strong risk factor contributing to poor outcomes in breast cancer and aggressive metastatic disease. The FS-specific attributes, which in extenso have been described earlier [29], clearly argue for this conclusion, namely,

- FS is prevalent in young populations;
- FS is more typical for females;
- FS symptoms appear early during the teenager period of life and moderate in the post menopause;
- Dysregulation/abnormalities of the cardio-vascular component characteristic for the FS strongly predispose the affected individuals to the chronic systemic hypoxic effects;
- At the molecular level, enhanced blood levels of endothelin-1 and metalloproteinases MMP-9 and MMP-2 have been described for the FS-affected individuals [3, 27, 29, 31] which at the same time is considered as a powerful prognostic biomarker panel for particularly poor outcomes in both breast cancer and metastatic disease [1, 3, 32].

The meaning of individual FS symptoms specifically for the BC patient cohort has been discussed in detail in the recently published article “Breast Cancer and Flammer Syndrome: Any Symptoms in Common for Prediction, Prevention and Personalised Medical Approach?” [28]. Current chapter emphasises a particular relevance of the FS symptoms for the metastatic disease in the BC patient cohort: all 15 symptoms demonstrate the prevalence in the metastatic BC versus BC-free reference group with a statistical significance (*p* ≤ 0.05) for seven symptoms as summarised in Table 3. Other symptoms, even being statistically non-significantly prevalent within this study, can be of great importance for the BC pathology and metastatic disease. Hence, symptom 3 (low blood pressure, *p* = 0.127) is particularly relevant for the premenopausal subgroup and may strongly contribute the cardio-vascular component characteristic for the FS as explained above. Another example is the following: symptom 4 (dizziness, *p* = 0.762) has been described earlier as being permanently present and stepwise worsening in BC followed by metastasis in the brain [20, 33]. Finally, the normal feeling of thirst (symptom 6, *p* = 0.103) is extremely important and if diminished (here two times in BC versus BC-free) plays a crucial role in the body dehydration and BC development [20].

As for the next steps promoting our knowledge and skills within this scientific area, the severity of the FS symptoms should be essentially correlated with the specific molecular profiles relevant for the metastatic BC development and progression. Contextually, individual levels of the hypoxia-inducible factor 1 (HIF-1) and vascular endothelial growth factor (VEGF)—both highly relevant for the progression of the aggressive metastatic disease [14, 34]—should be correlated with the FS phenotype. This research is expected, further, to bridge the FS phenotype with the metastatic disease in other cancer pathologies such as the prostate cancer [35].

Particular attention should be dedicated to the specific molecular profiles relevant for pain sensitivity (symptom 10) in BC and metastatic disease. The reason for that is the functional link between pain, chronic inflammation and wound healing, which, if impaired, may lead to aggressive cancer malignancies. The recently published article “Impaired Wound Healing: Facts and Hypotheses for Multi-professional Considerations in Predictive, Preventive and Personalised Medicine” [36] discusses potential relevance of the FS for altered wound healing and suggests mechanisms and provides innovative concepts in the field.

### Concluding remarks

The results presented here do substantially extend the currently existing knowledge regarding the “Seed and Soil” theory of metastasis clearly demonstrating that a strong predisposition of individuals at risk to form the systemic hypoxic pre-metastatic niches can be established a long time before breast malignancy is clinically manifested. The FS phenotype may strongly contribute to the aggressive metastatic disease. The exploitation of the acquired knowledge might be performed by creating innovative screening programmes. Contextually, we propose the FS questionnaire to be considered by family doctors (GPs) for its practical application to the general population, in order to improve the quality of primary care by developing novel, more effective approaches for the targeted prevention of metastatic BC. For that, the best appropriate 13 FS symptoms are grouped together within the cluster marked in green colour as summarised in Table 3. The relevance of the remaining two symptoms should be investigated additionally prior to considering their practical application, due to the high level of plurality amongst the BC patients demonstrated in the current study.

BC, breast cancer; BMI, body mass index; CTC, circulating tumour cells; ECM, extra-cellular matrix; FIA, fibro-adenoma; FS, “Flammer Syndrome”; GPs, general practitioners (family doctors); HER-2, human epidermal growth factor receptor 2; HIF-1, hypoxia-inducible factor 1; ТxNxМx, tumour size/number of affected lymph nodes/number of metastases; VEGF, vascular endothelial growth factor.
